# Hydrogen Is the Superior Nebulization Gas for Desorption and Electrospray Ionization

**DOI:** 10.1021/acs.analchem.4c03867

**Published:** 2024-09-22

**Authors:** Bincy Binny, George Joseph, Andre R. Venter

**Affiliations:** Department of Chemistry, Western Michigan University, Kalamazoo, Michigan 49008-5413, United States

## Abstract

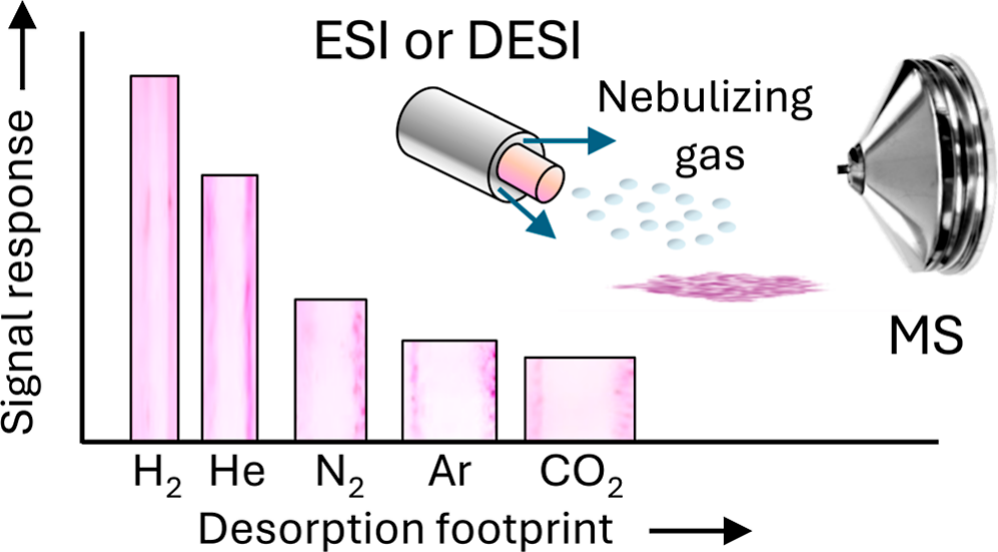

A previous comparative study between helium and nitrogen as nebulizing and desolvation gases in electrospray ionization (ESI) and desorption electrospray ionization (DESI) found that the signal responses of compounds of varying sizes and polarities were improved. Here, an expanded selection of nebulizing gases was evaluated to investigate mechanisms of improvement. The set of nebulizing gases included hydrogen, helium, nitrogen, argon, and carbon dioxide. Results indicate that the signal enhancements are achieved by gases lighter than nitrogen and that the previously described helium effects can be improved by using the more economical and sustainable hydrogen as a nebulizing gas. Additionally, H_2_ and He reduce the desorption footprint, which could be potentially useful in increasing the resolution of chemical imaging microscopy, especially since, despite the smaller footprint obtained using helium and hydrogen, higher signals are obtained compared to nitrogen.

## Introduction

Electrosonic spray ionization (ESSI) is a soft ionization technique that employs a traditional micro-ESI source with a supersonic nebulizing gas for efficient nebulization and desolvation.^1^ Similar ion sources are used for both electrospray ionization (ESI) and home-built desorption ionization (DESI) mass spectrometry (MS).^2^ Nebulization is aided with the use of a gas to create a fine mist of accelerated droplets that is charged by the applied potential difference between the nebulizer and the mass spectrometer inlet. N_2_ is typically used as a nebulizing gas in ESI and DESI.^2,3^ Oxygen,^4^ air,^5^ carbon dioxide, and ammonia^6^ were previously investigated for nebulizing gas with ESI. SF_6_ was reported to be useful for the suppression of discharges in ESI.^7,8^

In DESI-MS, the sample is external to the ionization source on a surface from where molecules are dissolved into a transient and localized solvent microlayer; analytes subsequently leave the surface in secondary droplets to be ionized through ESI processes. Javanshad et al. reported using helium-assisted desorption and ionization,^9^ where helium serves as a nebulizing gas in ESI and DESI. This approach has enabled the analysis of a broader range of compounds with varying polarities and sizes and provides signal improvements, especially for lower-polarity compounds.

In this paper, we aim to better understand the origin of improved signal responses observed with helium as a nebulizing gas in ESI and DESI by comparing He to other nebulizing gases such as H_2_, which is even lower in mass but diatomic; Ar, which also has a favorable ionization energy and is a noble gas; and the CO_2_ molecule, which is similar in mass to the Ar atom.

## Experimental Section

### Chemicals and Materials

High-purity nitrogen, helium, argon, hydrogen, and carbon dioxide (>99%) were purchased from Airgas (PA, USA). HPLC–MS-grade methanol (MeOH) and LC–MS-grade formic acid (FA) were purchased from Sigma-Aldrich (MO, USA). Milli Q water (18 MΩ cm^–3^) was obtained from a Thermo-Barnstead Water Polisher (Thermo Scientific, Waltham, MA, USA). Hydrocortisone, scopoletin, phenanthrene, equine muscle myoglobin, and methyl salicylate were purchased from Sigma-Aldrich (MO, USA). The SPLASH LIPIDOMIX mass spectrometry standard was purchased from Avanti polar lipids (AL, USA). Fused silica capillaries were purchased from Trajan Scientific (Ringwood, NSW, Australia). PTFE Omni Slide was purchased from Prosolia Inc. (IN, USA).

### Instrumentation

A linear ion trap LTQ mass spectrometer (Thermo Scientific, Waltham, MA, USA) was combined with a 3-dimensional translational stage (Purdue University, West Lafayette, IN, USA). A 2 cm extended ion transfer tube was purchased from Adaptas Scientific Instrument Services, MA, USA. Electrosonic sprayers and desorption sprayers were made using a coaxially fused silica capillary held in a Swagelok T-piece. The outer silica capillary for gas delivery was approximately 15 mm in length and had an inner diameter of 250 μm and an outer diameter of 360 μm. The inner capillary for solvent delivery had an inner diameter and an outer diameter of 50 and 150 μm, respectively. The inner capillary extended through the T-piece to the syringe pump for solvent or sample delivery.

### Sample Preparation

Samples were pneumatically sprayed on a glass surface using a nebulizer made of coaxial fused silica capillaries similar in size to those listed above. The sprayer was held in an orthogonal position, 2.0 cm above the surface. The stage moved at 200 μm/s. The flow rate and nebulizing gas pressure were 3 μL/min and 120 psi, respectively. The width of the resulting homogeneous sample lines was 1.0 ± 0.1 mm. Sample stock solutions were prepared at 40 μM, resulting in average surface concentrations of 10 pmol/mm^2^. For the PTFE surface, 3 μL of sample solutions at 50 μM was pipetted on the surface and vacuum-dried for 30 min for average surface concentrations of 20 pmol/mm^2^.

### DESI-MS and ESI-MS Parameters

The DESI sprayer was positioned at an angle of 54°. The optimized distances of the sprayer from the ion transfer tube and the surface were nominally 4 and 1 mm, respectively. The source temperature was set to 250 °C. The spray voltage was 4 kV. The flow rate and the gas pressure were 5 μL/min and 120 psi, respectively. The capillary voltage and tube lens voltage were optimized between 20–40 V and 90–175 V, respectively. For ESI experiments, the sample solutions were nebulized by the ESSI sprayer (same construction as the DESI source) toward the ion transfer tube at an angle of 44°, and the distance of the sprayer from the ion transfer tube was 7 mm, while all other conditions were maintained.

## Results and Discussion

**Figure 1:**
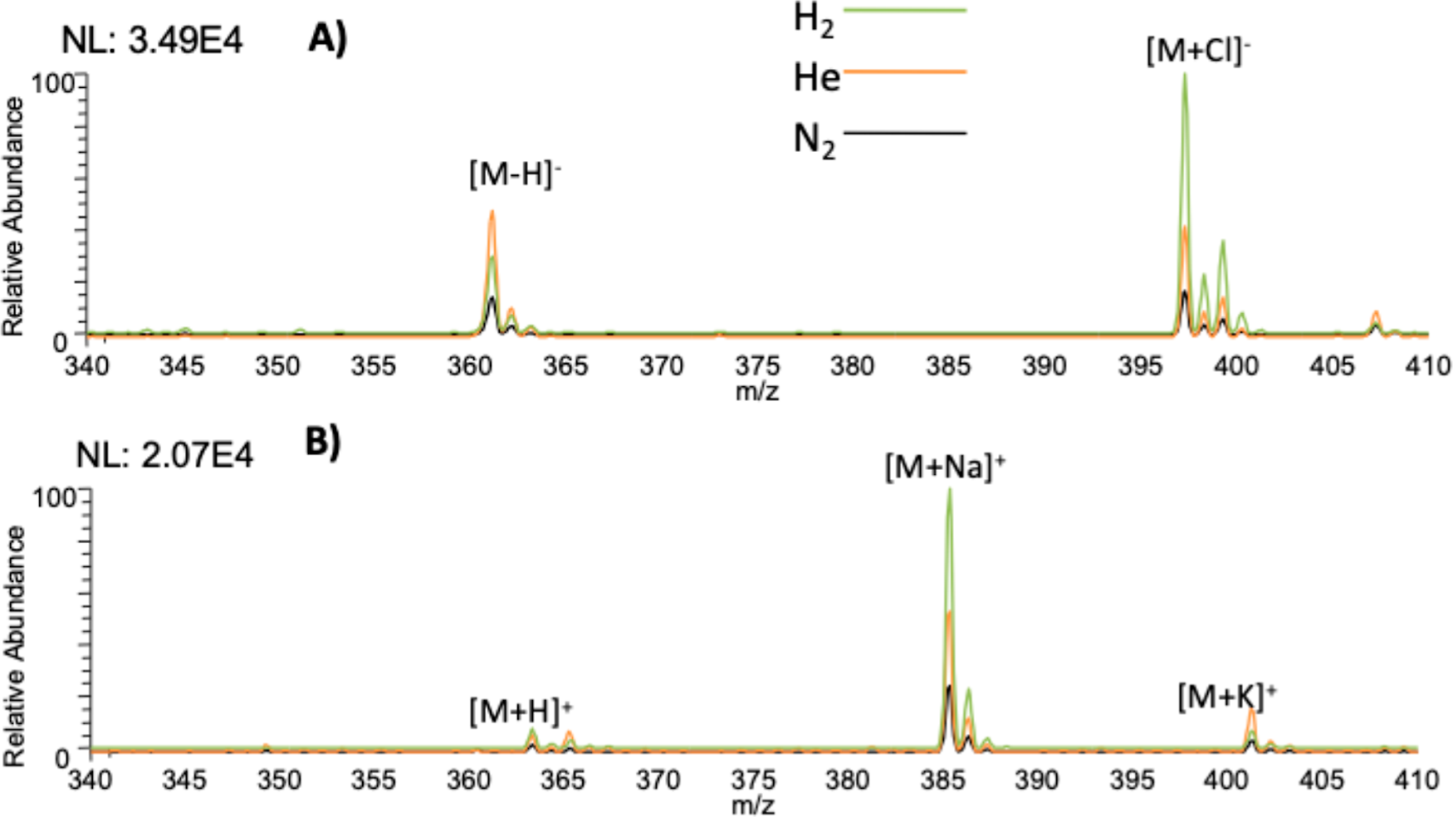
ESI-MS of nonpolar steroid hydrocortisone in 50% MeOH/H_2_O using different nebulizing gases in (A) negative mode and (B) positive mode of ionization. The normalization level (NL) indicates the absolute signal intensity.

It was hypothesized that with helium-assisted desorption and ionization,^9^ the helium nebulizing gas may generate ions through electrospray as well as by charge transfer from long-lived plasma-generated ion transfer reagents (APCI). On the other hand, it is also possible that the observed improvements in ion counts originate from improved hydrodynamic and thermal properties of helium that aid the nebulization and desolvation processes in ESI. Under the source conditions applied in this work, reactive species were not observed (Figures S1 and S2), and APCI-only ionizable compounds were not observed in the MS using ESI with helium nebulization. Further, a common feature of the ESI mechanism is the formation of alkali metal adduct ions such as Na^+^ and K^+^ in positive mode and Cl^–^ adduct formation in negative mode. Figure 1 shows the analysis of 10 μM hydrocortisone, a low-polarity steroid in 50% MeOH/H_2_O. Deprotonation and chloride adduction were observed in negative ionization mode (Figure 1A). Ionization in positive mode yielded sodium and potassium adduct ions in addition to protonated peaks, regardless of the nebulizing gas employed (Figure 1B). The signal response improved between two and four times when switching to helium or hydrogen in both positive and negative modes.

We conclude that it is unlikely that atmospheric pressure chemical ionization significantly contributes to the observed signal improvements under the employed conditions. This allows us to focus on nebulization, scattering, and evaporative aspects of the different nebulization gases in this article.

### Nebulization Gas Effects

**Figure 2:**
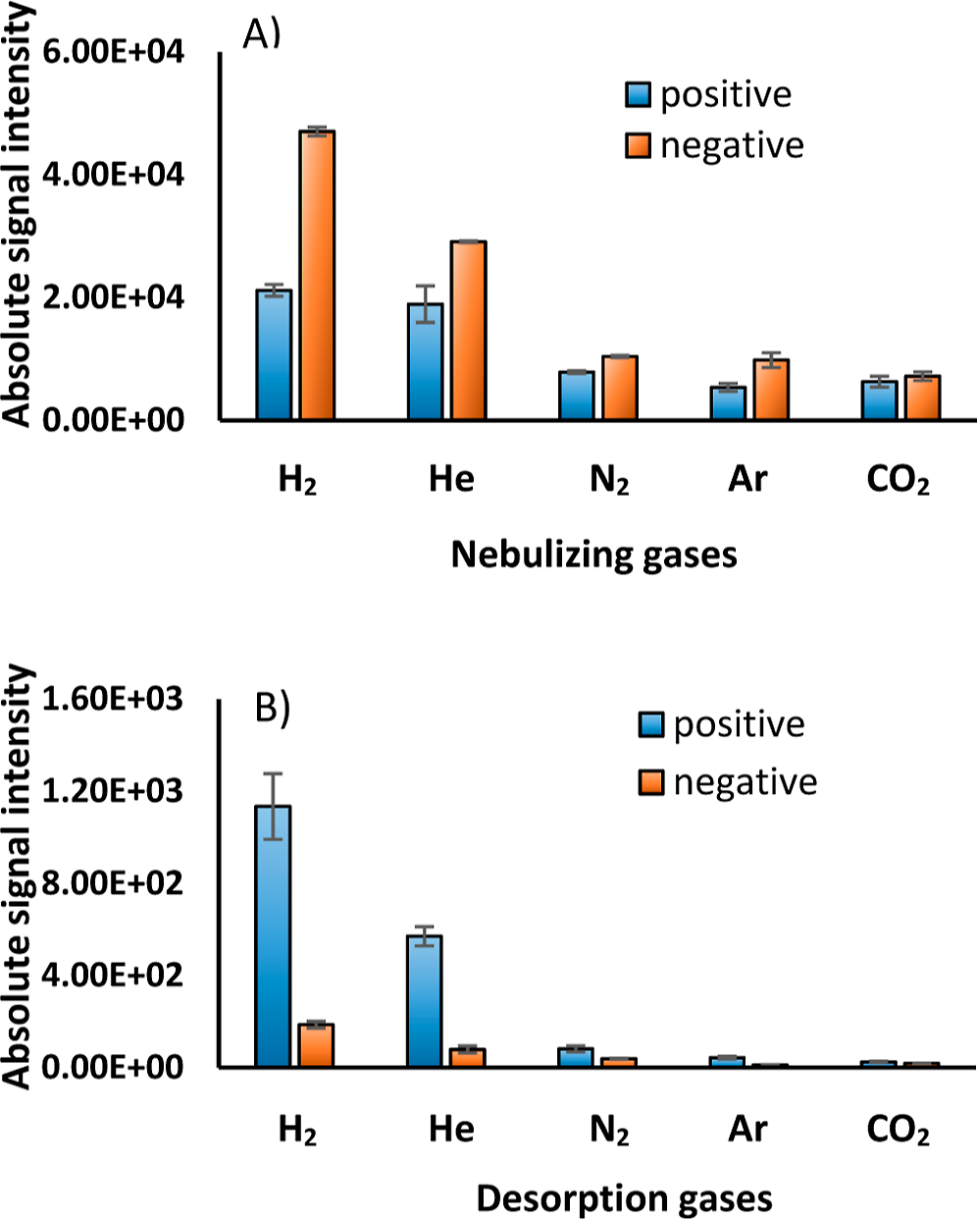
Signal response of hydrocortisone obtained using different nebulizing gases (N_2_, He, H_2_, CO_2_, and Ar) in positive mode and negative mode, indicated by blue and orange, respectively, in (A) ESI and (B) DESI.

The chemicophysical properties of nebulizing gases play an important role in the nebulization, evaporation, and ion transport processes in ESI and additionally also desorption in DESI. To investigate differences in nebulization, we compared He to H_2_, which is even lower in mass but diatomic; Ar, a noble gas and is monatomic, which is heavier than N_2_; and the CO_2_ molecule, which is similar in mass to the Ar atom. Figure 2A shows the signal responses obtained for ESI of 10 μM hydrocortisone in 50% MeOH/H_2_O using different nebulizing gases, and Figure 2B depicts the same for DESI. Representative mass spectra are provided in Figures S3 and S4. The reported intensity values in Figure 2 show the sum of the protonated and adducted species.

In ESI, switching the nebulization gas from N_2_ showed about 2.4 and 2.7 times improvement in the signal for the steroid for both lighter gases, He and H_2_, in positive ionization mode. Considering the relative energies of metastable hydrogen (11.8 eV)^10^ and helium (19.8 eV),^11^ this underscores the importance of nebulization and solvent evaporative effects under these conditions. CO_2_ and Ar, both heavier than N_2_, did not significantly change the intensities. In negative mode, ESI with hydrogen delivered a larger improvement (4.5×) compared to helium (2.8×) in signal improvements for hydrocortisone (Figure 22A). The scopoletin signal showed around two times improvement in the signal for both H_2_ and He in positive mode of ionization. Negative mode ionization of scopoletin provided 4.4 and 2.9 times improvement for H_2_ and He, respectively (Figure S5).

The methyl salicylate signal was also improved in negative mode ESI, where the deprotonated peak showed an improvement of approximately 2.5 times with either He or H_2_ as the nebulization gas relative to N_2_, as shown in Figure S6.

Curiously, in ESI, a larger signal was obtained for hydrocortisone in negative ionization, and the relative improvements were also larger, while the opposite is true for DESI-MS, where the larger signal and improvements were observed in positive mode. In the DESI-MS positive mode, signals increased by 7 and 14 times for helium and hydrogen, respectively. On the other hand, the negative mode improvements were 2.0 and 4.8 times, respectively.

### Desorption Effects in DESI

While DESI shares the same ionization mechanism as ESI, additional desorption and surface effects come into play. As expected, the signal intensities with DESI are lower than those for equivalent ESI samples. The sample surface used in this work, PTFE, is inherently negatively charged and likely reduces droplet–surface interactions when the droplets are also negatively charged. The electronegativity difference between silicon and oxygen is not as significant as that between fluorine and carbon, and glass has a less negative surface compared to that of PTFE. In addition, the relative permittivity of PTFE is at least half that of glass, meaning that the documented capacitative effects of DESI^12^ are also larger on PTFE than on glass. When changing from PTFE to glass, the positive mode still produced larger signals and improvements, as can be seen in Figure S7, where helium provided an improvement of 6.4× and hydrogen, 11.3×. In negative mode DESI-MS, the signals were lower, but the improvements approached those of positive mode, at 4.4× with He and 11.2× with hydrogen nebulization. Hypothetically, this indicates that the changes in droplet momentum when switching from N_2_ to the lighter gases are on the same order of magnitude as the differences in surface negative charge buildup between PTFE and glass. This would, however, need to be experimentally verified using a similar approach as in the work by Gao et al.^13^

Relative improvements correlate with the masses of the gases in a negative logarithmic dependence (Figure S8A) rather than atomic and molecular radii (Figure S8B). A lighter gas yielded a greater ion signal for samples, as seen in Figure 2B.

**Figure 3:**
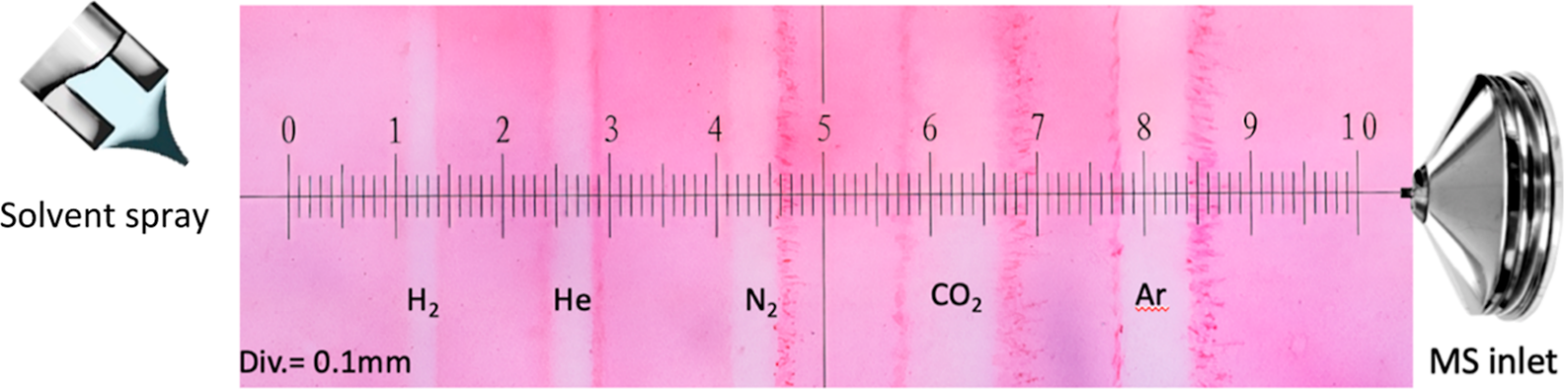
Photograph of the TerraSlate Laser Printer Copy paper uniformly covered in Rhodamine 6G after desorption by DESI using H_2_, He, N_2_, CO_2_, and Ar as nebulizing gases. The sample surface was overlaid with a calibration slide during photography, where the smaller divisions are equal to 100 μm.

To investigate the improved signal response with lower-mass gas-assisted desorption compared to regular DESI, we measured the differences in footprints obtained with different nebulization gases, as demonstrated in Figure 3. Desorption footprints have an ellipsoidal profile.^14^ We report the length of the major axis of the ellipse for the footprint as opposed to the minor axis. Desorption profiles obtained using CO_2_ and Ar were 900 and 600 μm, respectively, about two times larger compared with N_2_ with a desorption profile of 400 μm. The footprints obtained using He and H_2_ were smaller and 300 and 200 μm, respectively. Despite the smaller footprint obtained using helium and hydrogen, larger signals were obtained compared to nitrogen.

The electrosonic spray ionization source uses high pressure and large volumetric flow rates of nitrogen gas for efficient nebulization and droplet acceleration of charged microdroplets.^1^ Phase doppler particle anemometry (PDPA) measurements of droplets produced by nitrogen under ESSI conditions measured droplet size distributions with a median value of 5 μm and an average velocity of 100 m/s.^15^ Kinetic energy on impact of such droplets was estimated to be in the order of 500 MeV. In a different study using lower volumetric flow rates, droplet size and velocity distributions for helium and argon were compared in a study of pneumatic nebulizers. It was shown that helium produces 10% larger and 25% faster moving droplets with a more narrow velocity distribution compared to argon.^16^ Larger droplets were also reported for helium in other studies; however, here faster velocities were reported for argon.^17,18^ The conflicting results for droplet velocity between the two studies emphasize the crucial role of the nebulizer design.^19^

Helium and hydrogen likely produce faster moving and larger inbound droplets that would deliver a more energetic impact on the microlocalized liquid layer on the sample surface. This would efficiently release smaller secondary droplets from the surface liquid layer, with enough kinetic energy to be lifted off from the surface with a better trajectory toward the ion transfer tube. Second, due to the low mass of hydrogen molecules and helium atoms, the inbound spray, containing primary droplets and Coulombic explosion products (progeny droplets and ions), would experience less scattering, leading to a tighter desorption footprint.

### Desolvation Effects

The thermal conductivities of hydrogen and helium, 186.9 and 156.7 mW/m K, respectively, are significantly higher than those of argon and nitrogen with thermal conductivities of 17.9 and 26.0 mW/m K, respectively.^17,20,21^ The higher thermal conductivities in helium and hydrogen facilitate more efficient droplet evaporation and analyte desolvation, even though larger primary droplets are produced.^16^

### Ion Transmission Effects

Transmission of ions and droplets through the ion transfer tube will also be influenced by the various nebulization gases. Air flow through an ion transport tube has been shown to be at least transitionary and likely turbulent.^22^ Turbulent flow in an ion transfer tube is dependent on the molar mass of the gas atom/molecule and its dynamic viscosity.^22^ Based on gas atom/molecule weights and dynamic viscosity values for the gases, hydrogen and helium would have Reynolds numbers around seven times lower than those of nitrogen and argon, while for carbon dioxide, it would double. The lower Reynold numbers of hydrogen and helium could indicate that a significant contribution to the increased signal intensities is likely due to a more laminar flow and, hence, improved ion transmission.

The volumetric flow of helium and hydrogen is measurably greater than heavier gases N_2_, Ar, and CO_2_ at atmospheric pressure after a restrictor at the same nominal input pressure. Additionally, the supersonic expansion of gas on reaching the first vacuum region of the mass spectrometer will lead to high collision frequencies in the first vacuum region of the instrument, facilitating efficient desolvation and collisional ion cooling.^23,24^ Lighter background gases, such as helium or hydrogen, causes less ion scattering, there by improving ion sampling efficiency.^25^ Simultaneously, CID, usually a phenomenon to be avoided during ion transfer, is minimized by using lighter bath gases, and hydrogen molecules also have a vibrational mode capable of additional cooling relative to helium.^26^

### Hydrogen: A Better Nebulization Gas

Although helium-assisted nebulization proved beneficial for a variety of analytes,^9^ helium is a limited natural resource with an increasing demand. It is therefore important to find an alternative or reduce the consumption of helium. Hydrogen, on the other hand, is a renewable resource and significantly less expensive.

**Figure 4:**
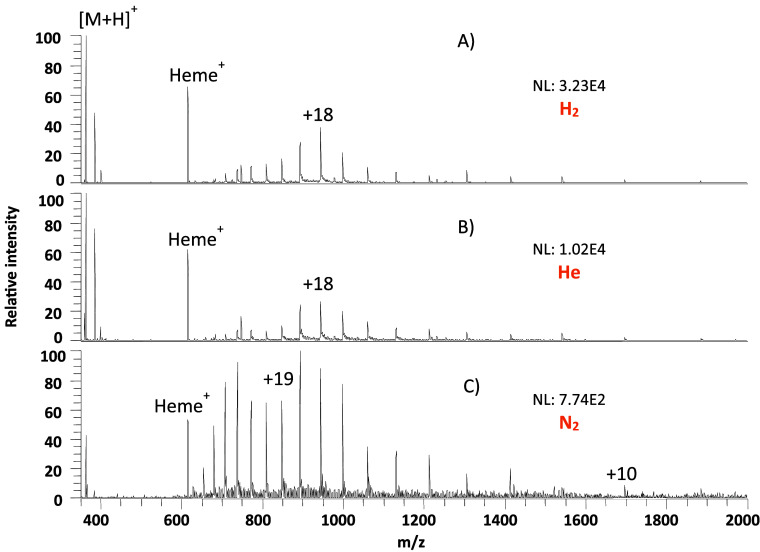
DESI-MS spectra of 10 pmol/mm^2^ each of hydrocortisone and myoglobin on glass microscope slides desorbed using 0.2% formic acid in 50% MeOH/H_2_O as the desorption solvent with (A) hydrogen, (B) helium, and (C) nitrogen as nebulizing gases. The normalization level (NL) indicates the absolute signal intensity.

The use of helium and hydrogen as nebulizing gases for the simultaneous detection of proteins and small molecules in desorption electrospray ionization is illustrated in Figure 4.

An equimolar sample mixture of myoglobin and hydrocortisone was spray-deposited on a glass microscopic slide and desorbed using 50% MeOH/H_2_O containing 0.2% FA as an additive. The signal response of the steroid improved between 15 and 20 times on switching from N_2_ to He while still allowing the detection of multiply charged apo-myoglobin and improving its signal about five times. Switching to H_2_ provided an additional three times improvement for hydrocortisone and six times for apo myoglobin, or 45 and 30 times relative to nitrogen, respectively.

**Figure 5:**
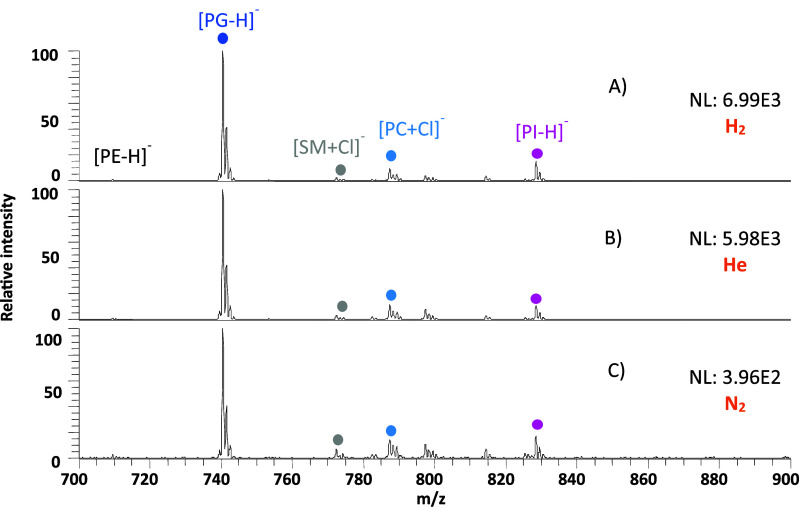
DESI-MS spectrum of the lipid splash mix on a PTFE surface desorbed using 50% ACN/DMF as the desorption solvent using (A) hydrogen, (B) helium, and (C) nitrogen in negative ionization mode. The normalization level (NL) indicates the absolute signal intensity.

**Figure 6:**
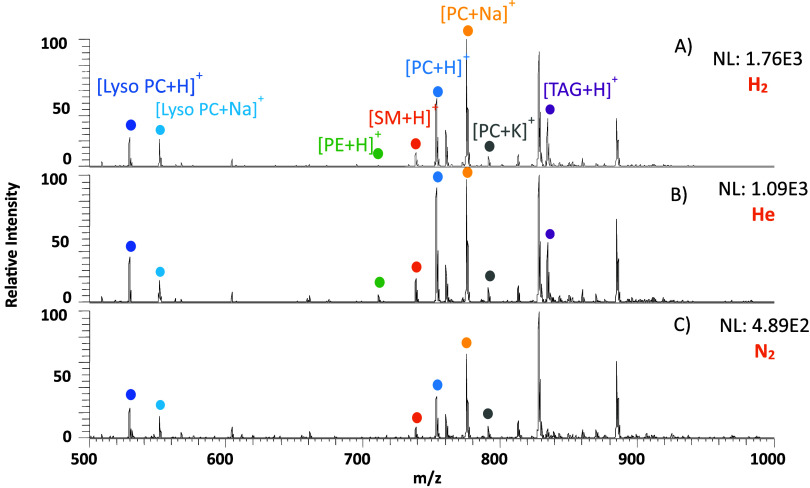
DESI-MS spectrum of the lipid splash mix on a PTFE surface desorbed using 50% ACN/DMF as the desorption solvent using (A) hydrogen, (B) helium, and (C) nitrogen in positive ionization mode. The normalization level (NL) indicates the absolute signal intensity.

DESI-MS serves as a useful tool for imaging thin sections of biological tissues, allowing detection of lipids and metabolites.^27^ The SPLASH LIPIDOMIX lipid standard was deposited as spots on PTFE using a micropipette. Figure 5 shows the detection of PG, SM, and PI lipids from the lipid standard using 50% ACN/DMF as the desorption solvent in negative ionization mode. Deprotonated ions for those lipids that ionize in negative mode showed improvements in the signal response between 10 and 20 times using He and H_2_ (Table S1). In positive mode, lipid signals of lyso-PC, PC, PI, PS, and SM were seen to improve between two and five times for He and slightly more with H_2_ on switching from N_2_. Protonated, sodiated, potassiated, and ammonium- and acetate-adducted ions were observed (Table S2). Figure 6 illustrates the use of hydrogen and helium for lipid analysis in positive ionization mode.

### Cautionary Notes

Hydrogen poses an explosion risk when present at concentrations between 4 and 75% in air at atmospheric pressure and in the presence of sparks and flames; thus, safety precautions and good ventilation are recommended.

Further, while the other gases investigated in this study are inert, hydrogen can behave as a strong reducing agent under certain conditions. Its use as a nebulizing gas might compromise the integrity of analytes that could react at the liquid/gas interface or in the gas phase after droplet evaporation, even in the short time frame of the ionization process. This was not the case for any of the compounds investigated in this study, but the risk of such a chemical reaction would need to be evaluated for analytes susceptible to reduction reactions.

## Conclusions

Although helium has the potential to enhance ion production by activating an additional APCI mechanism under the conditions studied, APCI was shown to not significantly contribute to the observed signal enhancements. This is based on (1) the inability of helium-assisted desorption and spray ionization to effectively ionize very nonpolar compounds such as phenanthrene, (2) the prevalence of alkali metal and chloride adducts on ions, and (3) the lack of a noticeable increase in charge transfer reagent species. This allowed us to study the improvements in nebulization, desorption, desolvation, and ion transport properties during the use of nontraditional nebulization gases. Signal increases scale inversely with the masses of the nebulizing gases, and the best results were obtained with hydrogen. Helium and hydrogen also have excellent thermal conductivities and low viscosities, leading to increased ability to desolvate and transfer ions into the mass analyzer. Helium as a nebulizing gas in electrospray ionization and desorption proved advantageous for the analysis of a variety of sizes and polarities of compounds. However, helium is a limited natural resource, and it is important to find alternatives and reduce its consumption. Renewable, cost-efficient hydrogen proves to be an even better alternative for nebulization and desorption, providing even higher signal improvements for low-polarity analytes, proteins, and lipids.

## Supporting Information Available

Mass spectrum for all analyzed compounds in positive and negative ionization modes in ESI-MS and DESI-MS, low mass region of ESI-MS and APCI-MS, analyses of additional compounds and their relative improvements, and plots of signal response with molecular or atomic masses and radii of different nebulizing gases (PDF)
