# Supplementary material for: Hydrogen Is the Superior Nebulization Gas for Desorption and Electrospray Ionization
Source: Anal Chem. 2024 Sep 22;96(39):15773–9. doi: 10.1021/acs.analchem.4c03867 (PMC11447664; doi:10.1021/acs.analchem.4c03867)
Supplement: Supplementary file 1 — ac4c03867_si_001.pdf [file ac4c03867_si_001.pdf]

Supplemental Information

Hydrogen Is the Superior Nebulization Gas for Desorption and Electrospray ionization

Bincy Binny, George Joseph, Andre R. Venter*

Department of Chemistry, Western Michigan University, Kalamazoo, Michigan, 49008, United States

andre.venter@wmich.edu

Table of contents

Figure S1. Comparison of low mass region of ESI-MS and APCI-MS for N_2, H_2, He, Ar and CO_2 as nebulising gas in positive mode of ionization shows lack of reactive species obtained by ESI.

Figure S2. Comparison of the low mass region of ESI-MS and APCI-MS for N_2, H_2, He, Ar and CO_2 as nebulising gas in negative mode of ionization shows lack of reactive species obtained by ESI

Figure S3. Mass spectra obtained for hydrocortisone using different nebulization gases in positive and negative mode ESI-MS.

Figure S4. Mass spectra obtained for hydrocortisone using different nebulization gases in positive and negative mode DESI-MS.

Figure S5. ESSI-MS of a relatively non polar compound, scopoletin, using different nebulizing gases in positive and negative modes of ionization.

Figure S6. Comparison of signals obtained for methyl salicylate using N_2, H_2 and He in ESI-MS.

Figure S7. Signal response obtained for DESI-MS of hydrocortisone on microscopic glass surface in positive and negative mode of ionisation.

Figure S8. Plot of improvements in signal for different gases compared to N_2 vs atomic/ molecular mass and radii of gases.

Table S1. Signal responses of lipids from Lipids Splash mix using different nebulising gases in negative mode of ionisation and improvement in signals obtained using He and H_2 compared to N_2

Table S2. Signal responses of lipids from Lipids Splash mix using different nebulising gases in positive mode of ionisation, improvement in signals obtained using He and H_2 compared to N_2 and observed adduct ions.

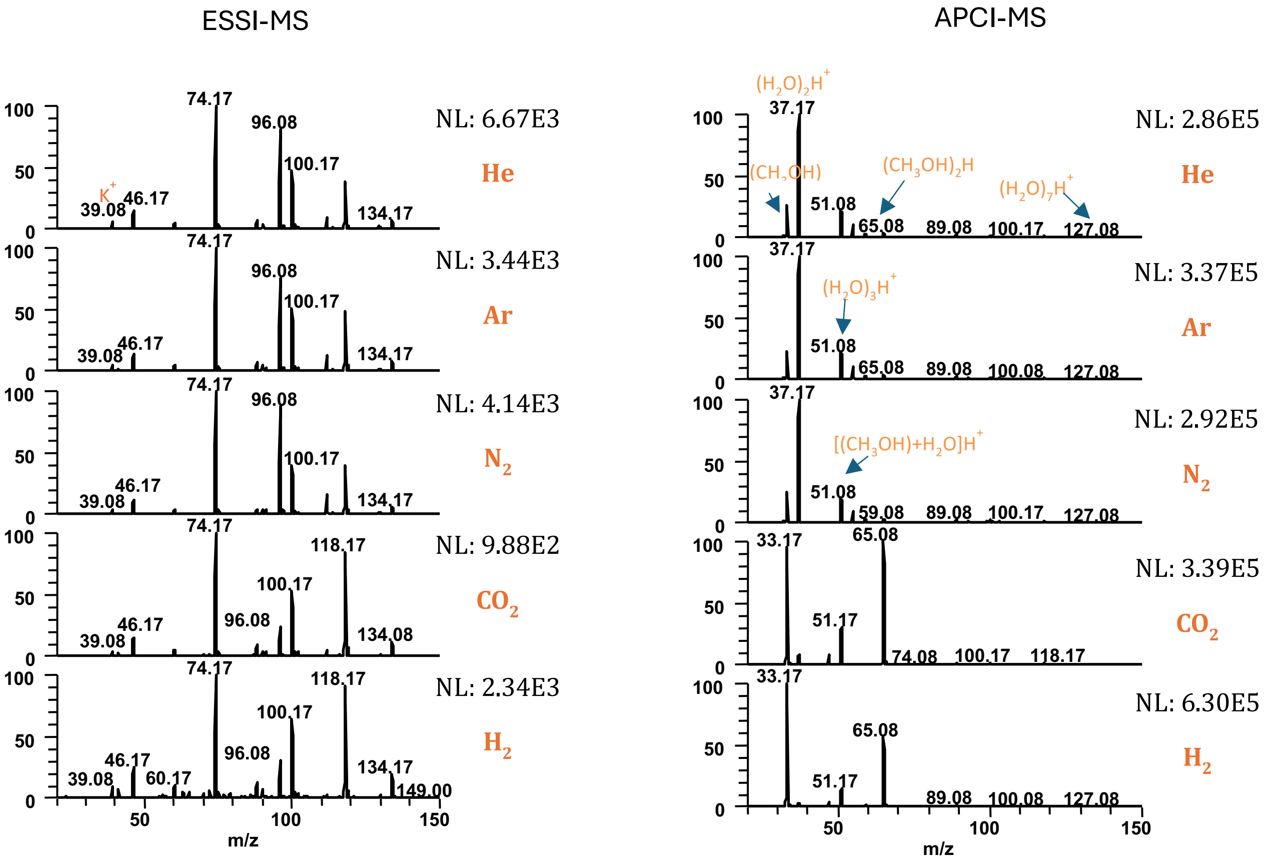

Figure S1: ESSI-MS and APCI-MS of 50% MeOH: H_2O in positive mode of ionisation using different nebulizing gases.

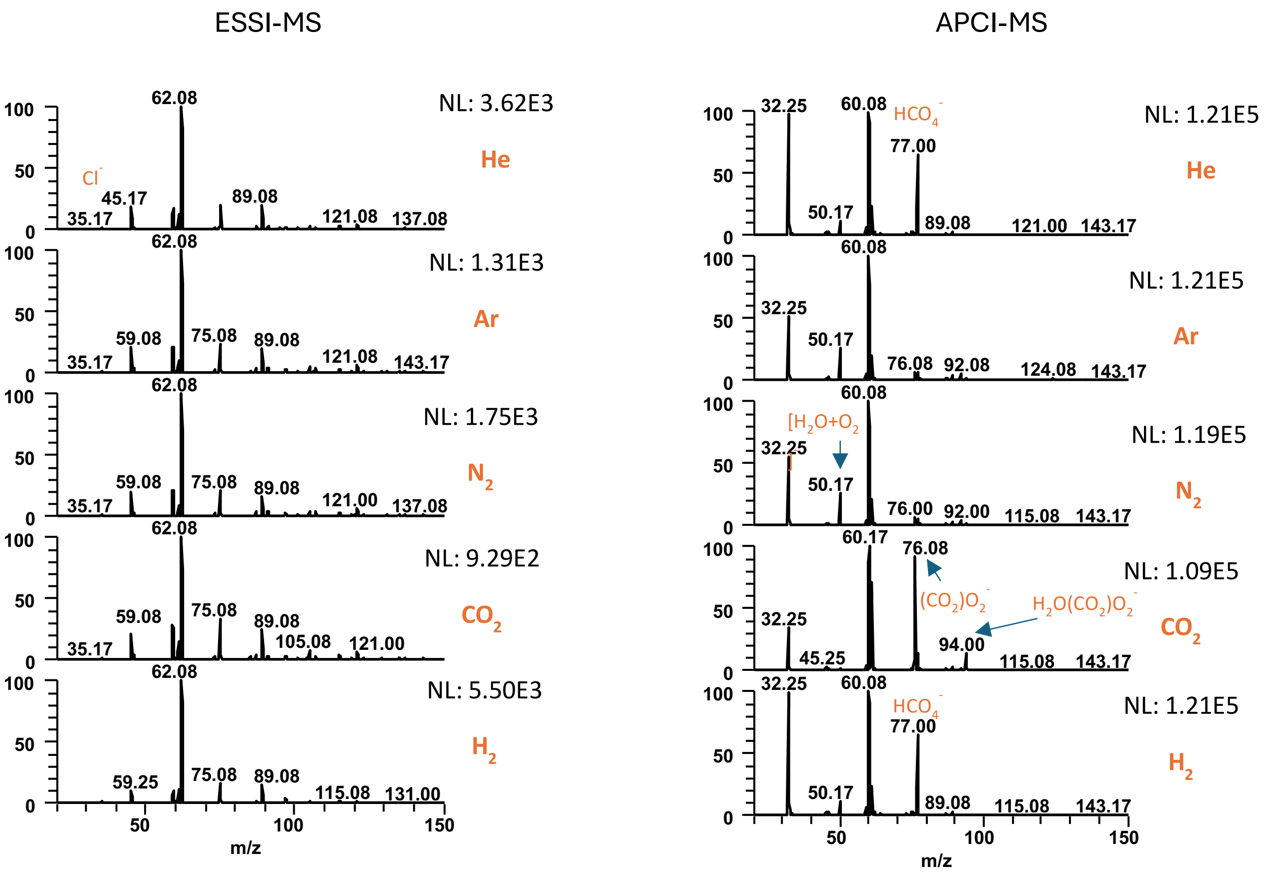

Figure S2: ESSI-MS and APCI-MS of 50% MeOH: H_2O in negative mode of ionisation using different nebulizing gases.

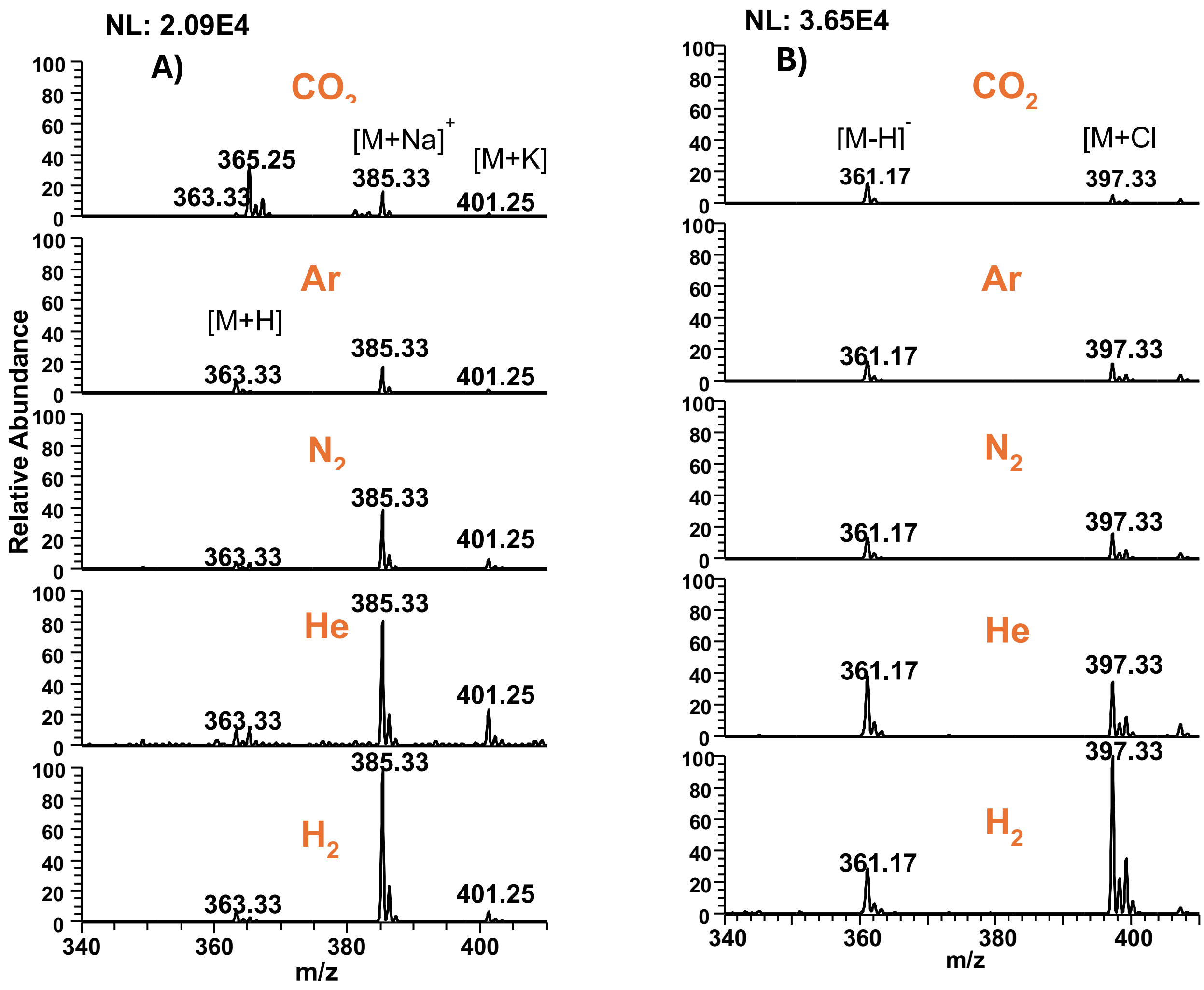

Figure S3. Mass spectra obtained for hydrocortisone using different nebulization gases in A) positive and B) negative mode in ESI-MS.

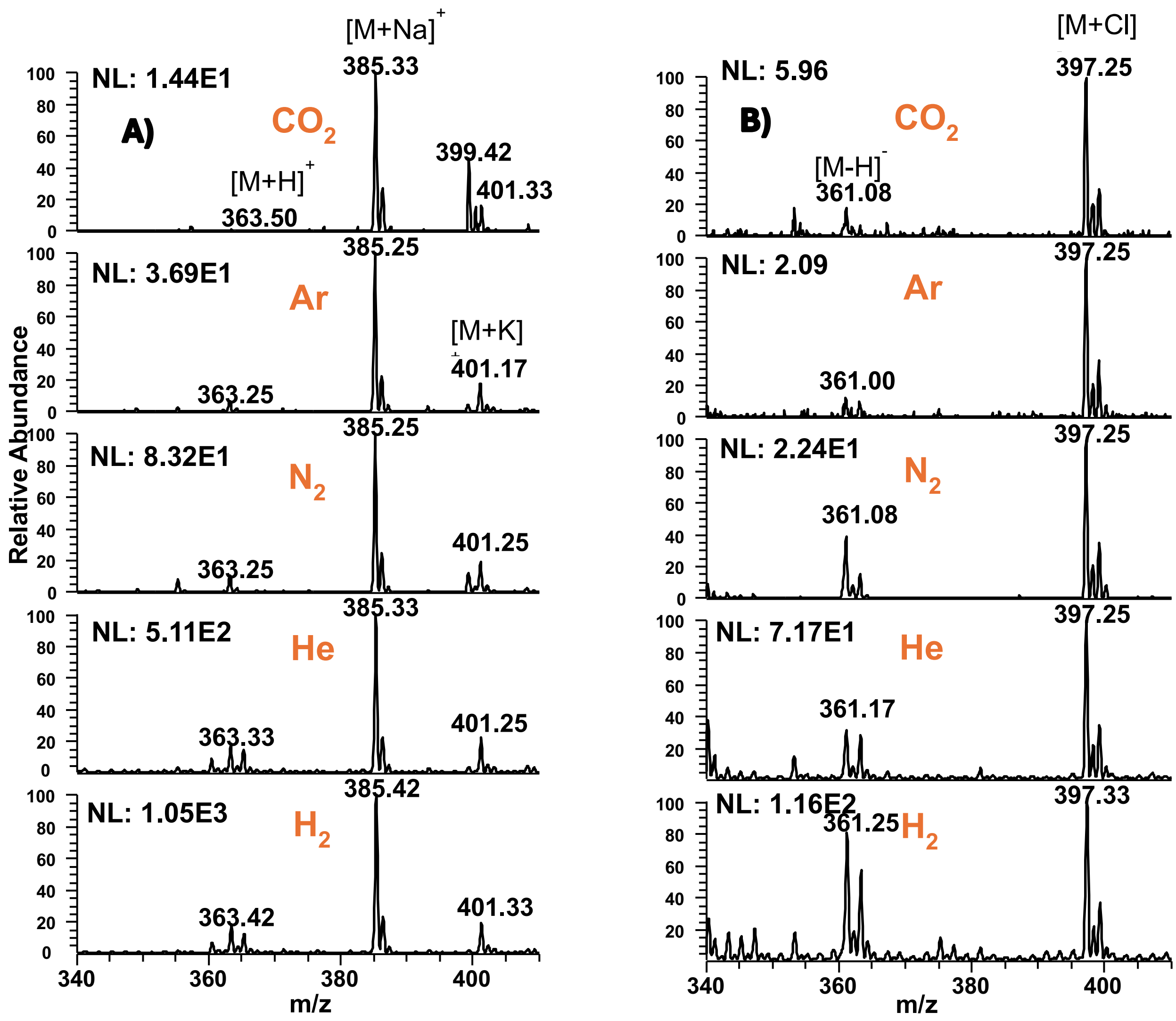

Figure S4. Mass spectra obtained for hydrocortisone using different nebulization gases in A) positive and B) negative mode in DESI-MS

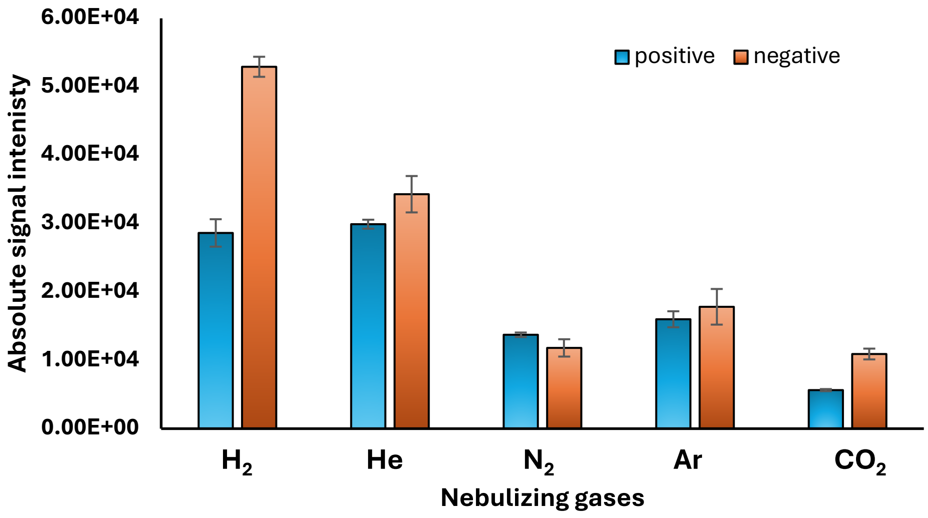

Figure S5: ESI-MS of 10 µM scopoletin using different nebulising gases in positive and negative ionisation.

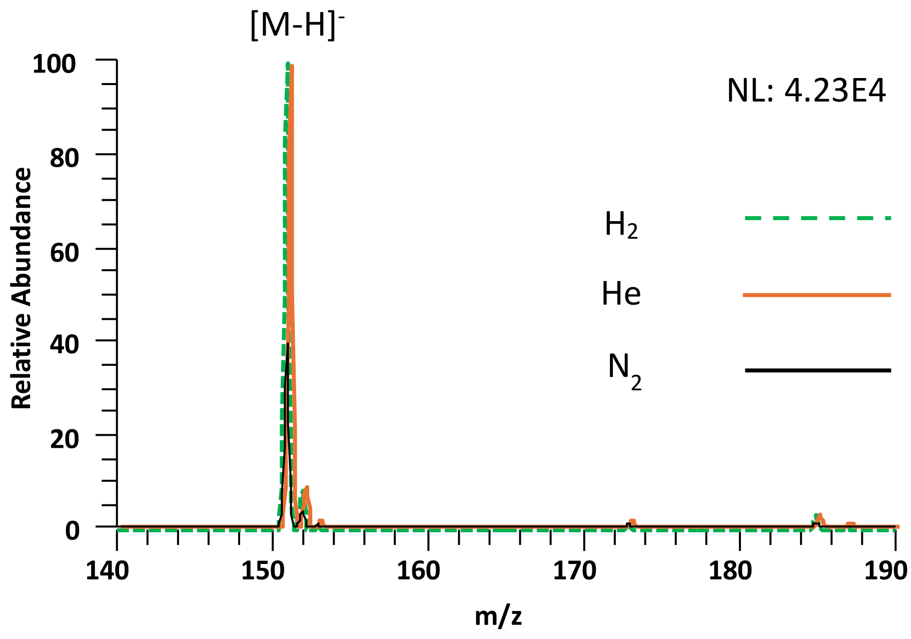

Figure S6: ESI-MS of methyl salicylate in 50% MeOH: H_2O in negative mode of ionisation using nitrogen (black), helium(orange) and hydrogen (green).

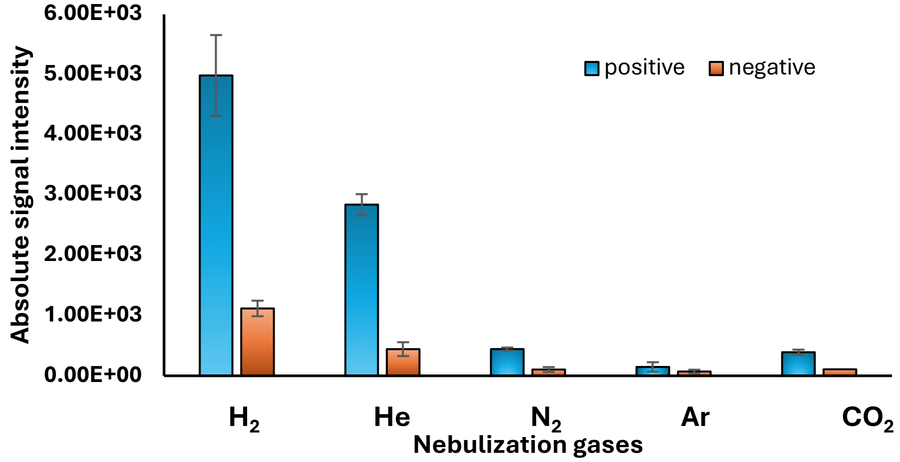

Figure S7: DESI-MS of 20 pmol/mm^2 hydrocortisone on a glass surface in positive and negative mode using different nebulising gases.

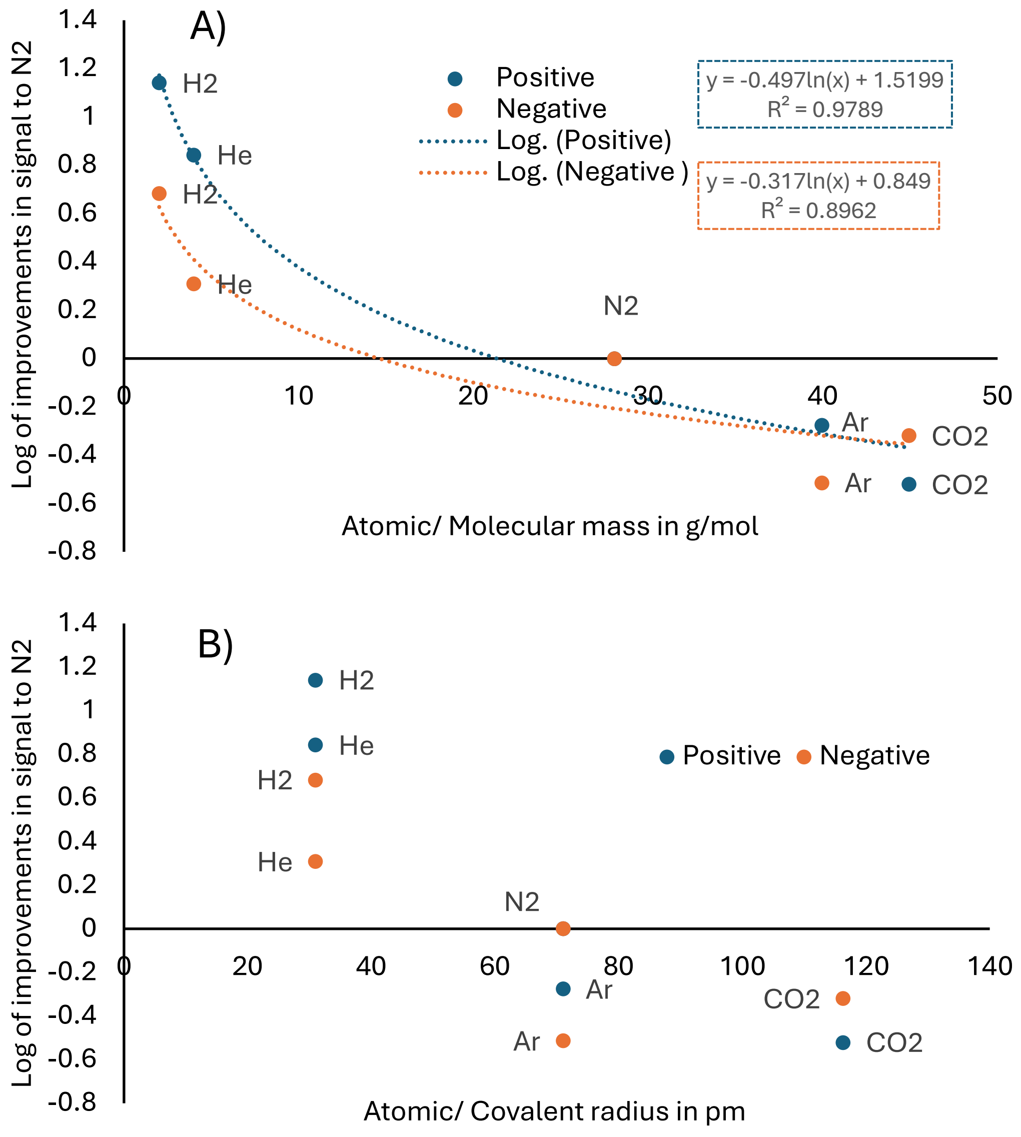

Figure S8: Plot of log of relative improvements in signal obtained using different nebulising gas with N_2 v/s atomic/ molecular mass (panel A), atomic/ covalent radius in pm (panel B) in DESI for hydrocortisone in positive and negative modes indicated by blue and orange respectively.

Table S1: Ionization of lipids in negative mode. "x" indicates ions observed for lipids.

Lipids	Adducts		Sum of intensity			He/N_2	H_2/N_2
			N_2	He	H_2		
15:0-18:1(d7) PC	[M-H]-		5.58E1	6.77E2	7.00E2	12.1	12.5
	$[M+Cl]^-$	x					
18:1(d7) lyso PC	[M-H]-		1.61E1	1.48E2	1.18E2	9.2	7.3
	$[M+Cl]^-$	x					
15:0-18:1(d7) PE	[M-H]-	x	4.39	8.80	8.73	2.0	2.0
	$[M+Cl]^-$						
18:1(d7) lyso PE	[M-H]-	x	3.40E1	5.09E2	3.88E2	15.0	11.4
	$[M+Cl]^-$						
15:0-18:1(d7) PG	[M-H]-	x	3.67E2	6.66E3	7.25E3	18.1	19.8
	$[M+Cl]^-$						
15:0-18:1(d7) PI	[M-H]-	x	6.08E1	6.98E2	1.38E3	11.5	22.7
	$[M+Cl]^-$						
15:0-18:1(d7) PS	[M-H]-	x	3.07E1	3.45E2	4.30E2	11.2	14.0
	$[M+Cl]^-$	x					
15:0-18:1(d7)-15:0 TAG	[M-H]-				1.08E1		
	$[M+Cl]^-$	x					
MAG	[M-H]-	x		1.26E1	5.49		
	$[M+Cl]^-$						
d18:1-18:1(d9) SM	[M-H]-		2.05E1	1.84E2	2.01E2	9.0	9.8
	$[M+Cl]^-$	x					

Table S2: Ionization of lipids in positive mode. "x" indicates adduct ions observed for lipids.

Lipids	Adducts		Sum of intensity			He/N_2	H_2/N_2
			N_2	He	H_2		
15:0-18:1(d7) PC	$[M+H]^+$	x	6.49E2	2.31E3	2.54E3	3.5	3.9
	$[M+NH_4]^+$						
	$[M+AcO]^+$						
	$[M+Na]^+$	x					
	$[M+K]^+$	x					
18:1(d7) lyso PC	$[M+H]^+$	x	1.57E2	4.60E2	3.99E2	2.9	2.5
	$[M+NH_4]^+$						
	$[M+AcO]^+$						
	$[M+Na]^+$						
	$[M+K]^+$						
18:1(d7) lyso PE	$[M+H]^+$				8.47E1		
	$[M+NH_4]^+$						
	$[M+AcO]^+$						
	$[M+Na]^+$	x					
	$[M+K]^+$						
15:0-18:1(d7) PI	$[M+H]^+$	x	6.67E1	1.76E2	1.89E2	2.6	2.8
	$[M+NH_4]^+$						
	$[M+AcO]^+$	x					
	$[M+Na]^+$						
	$[M+K]^+$						
15:0-18:1(d7) PS	$[M+H]^+$	x	1.17E2	3.27E2	3.03E2	2.8	2.6
	$[M+NH_4]^+$						
	$[M+AcO]^+$	x					
	$[M+Na]^+$						

	$[M+K]^+$						
15:0-18:1(d7)-15:0 TAG	$[M+H]^+$		3.85E2	1.03E3	1.21E3	2.7	3.1
	$[M+NH_4]^+$	x					
	$[M+AcO]^+$	x					
	$[M+Na]^+$	x					
	$[M+K]^+$	x					
d18:1-18:1(d9) SM	$[M+H]^+$	x	5.43E1	6.43E2	5.93E2	11.8	10.9
	$[M+NH_4]^+$	x					
	$[M+AcO]^+$						
	$[M+Na]^+$	x					
	$[M+K]^+$	x					
Cholesterol-d7	$[M+H]^+$		6.73E1	2.33E2	1.42E2	3.5	2.1
	$[M+NH_4]^+$						
	$[M+AcO]^+$						
	$[M+Na]^+$						
	$[M+K]^+$	x					
